# The molecular landscape of high-risk early breast cancer: comprehensive biomarker analysis of a phase III adjuvant population

**DOI:** 10.1038/npjbcancer.2016.22

**Published:** 2016-07-13

**Authors:** Timothy R Wilson, Jianjun Yu, Xuyang Lu, Jill M Spoerke, Yuanyuan Xiao, Carol O’Brien, Heidi M Savage, Ling-Yuh Huw, Wei Zou, Hartmut Koeppen, William F Forrest, Jane Fridlyand, Ling Fu, Rachel Tam, Erica B Schleifman, Teiko Sumiyoshi, Luciana Molinero, Garret M Hampton, Joyce A O’Shaughnessy, Mark R Lackner

**Affiliations:** 1 Department of Oncology Biomarker Development, Genentech Inc., South San Francisco, CA, USA; 2 Department of Biostatistics, Genentech Inc., South San Francisco, CA, USA; 3 Department of Research Pathology, Genentech Inc., South San Francisco, CA, USA; 4 Department of Bioinformatics, Genentech Inc., South San Francisco, CA, USA; 5 US Oncology, Dallas, TX, USA; 6 Baylor Sammons Cancer Center, Dallas, TX, USA; 7 Texas Oncology, Dallas, TX, USA

## Abstract

Breast cancer is a heterogeneous disease and patients are managed clinically based on ER, PR, HER2 expression, and key risk factors. We sought to characterize the molecular landscape of high-risk breast cancer patients enrolled onto an adjuvant chemotherapy study to understand how disease subsets and tumor immune status impact survival. DNA and RNA were extracted from 861 breast cancer samples from patients enrolled onto the United States Oncology trial 01062. Samples were characterized using multiplex gene expression, copy number, and qPCR mutation assays. HR^+^ patients with a *PIK3CA* mutant tumor had a favorable disease-free survival (DFS; HR 0.66, *P*=0.05), however, the prognostic effect was specific to luminal A patients (Luminal A: HR 0.67, *P*=0.1; Luminal B: HR 1.01, *P*=0.98). Molecular subtyping of triple-negative breast cancers (TNBCs) suggested that the mesenchymal subtype had the worst DFS, whereas the immunomodulatory subtype had the best DFS. Profiling of immunologic genes revealed that TNBC tumors (*n*=280) displaying an activated T-cell signature had a longer DFS following adjuvant chemotherapy (HR 0.59, *P*=0.04), while a distinct set of immune genes was associated with DFS in HR^+^ cancers. Utilizing a discovery approach, we identified genes associated with a high risk of recurrence in HR^+^ patients, which were validated in an independent data set. Molecular classification based on PAM50 and TNBC subtyping stratified clinical high-risk patients into distinct prognostic subsets. Patients with high expression of immune-related genes showed superior DFS in both HR^+^ and TNBC. These results may inform patient management and drug development in early breast cancer.

## Introduction

Breast cancer is a heterogeneous disease that is categorized clinically by immunohistochemical (IHC) staining of the three receptors; estrogen receptor (ER), progesterone receptor (PR), and the human epidermal growth factor receptor-2 (ERBB2/HER2).^1^ Seminal studies in the early 2000s demonstrated that gene expression signatures could classify breast cancers into distinct and reproducible molecular subgroups.^2–5^ In essence, breast cancer can be molecularly classified into luminal A and luminal B subgroups that are mostly comprised of hormone-receptor-positive (HR^+^) breast cancers; a basal-like subgroup that is mostly comprised of triple-negative breast cancers (TNBC); a HER2-enriched subgroup that is mostly comprised of HER2^+^ breast cancers and a normal-like subgroup that has been proposed to be mostly comprised of the contaminating tumor-surrounding stroma.^6^ PAM50 predicted subtypes within a defined IHC subgroup have prognostic implications, in that the luminal A subgroup has a better prognosis than the luminal B subtype. More recently, Prat *et al.* demonstrated that the HER2-enriched subgroup within HER2^+^ patients had a better outcome to trastuzumab-based therapy when compared with the non-HER2-enriched subgroup.^7^

Large genomic analyses have provided crucial insights into the genetic landscape of breast cancer.^8–14^ For example, a high prevalence of *PIK3CA* mutations and cyclin D1 amplification are observed in the luminal subtypes, whereas a high prevalence of mutation in *TP53* and alterations in DNA repair enzymes are observed in the basal-like subtype of breast cancer. These findings suggest that breast cancer is a complex and heterogeneous disease wherein distinct subtypes have diverse biological drivers. Recently, Curtis *et al*.^8^ demonstrated that breast cancer can be further subdivided into 10 subtypes based on the composite DNA copy number and RNA gene expression signatures, each of which have prognostic implications. Furthermore, using microarray data, Lehmann *et al.* ^15^ were able to identify six molecular subtypes within TNBC, each with prognostic implications. These observations highlight the heterogeneity that exists even within IHC-defined breast cancer subtypes. Interestingly, in the study by Lehmann, clinically actionable targets could be identified within each molecular subtype, suggesting that future therapeutic studies in TNBC should be conducted in biomarker-defined populations. Finally, recent advances in the area of cancer immunotherapy have shown that tumors with tumor infiltrating lymphocytes (TILs) have a higher pathological complete response (pCR) rate in the neoadjuvant setting, especially in TNBC and HER2^+^ cancers,^16–19^ suggesting that immunologically active tumors are more likely to achieve an optimal clinical response to anti-cancer therapies.

Clinical studies that assess the efficacy of experimental agents commonly characterize patients based on ER, PR, and HER2 IHC status and generally do not incorporate molecular subtype information into treatment decisions. In addition, most of the large-scale genomic profiling efforts published to date have focused on heterogeneous patient populations rather than the defined patient subsets that are often enrolled in clinical trials. The USO1062 phase III trial enrolled 2,611 high-risk early breast cancer patients defined by tumor size, nodal status and/or negativity for hormone receptors (T1–3, N1–2, M0; or T>2 cm, N0, M0; or T>1 cm, N0, M0 and both ER- and PR-negative), who were randomized to standard anthracycline/taxane chemotherapy with or without the addition of capecitabine. After a median follow-up of 6.4 years, 346 disease-free survival (DFS) events were observed and no improvement in DFS was observed (hazard ratio (HR), 0.84; 95% confidence interval, 0.68 to 1.04; *P*=0.1136).^20^ Little is known about the molecular landscape in a clinically defined high-risk population, therefore we aimed to define the impact of tumor heterogeneity on therapeutic benefit and overall prognosis in this patient population. We molecularly profiled 861 patients’ primary breast cancers using an 800-gene expression panel, a 35-gene copy-number alteration (CNA) panel and a *PIK3CA* mutation assay. We found that molecularly defined patient subsets showed differences in outcome following treatment with adjuvant chemotherapy and that the breast cancer subtypes contain genomic alterations that provide new therapeutic hypotheses.

## Results

### Molecular profiling of the USO 01062 patient population

To profile the molecular heterogeneity of patients enrolled onto this study, we developed a custom 800-gene expression panel that is comprised of various gene and pathway signatures relevant to breast cancer biology, a 35-gene CNA panel and a targeted mutation panel. Of the 2611 intent to treat population (ITT) enrolled onto the study, 1,539 patients had tissue available for exploratory biomarker analysis (biomarker evaluable population, BM). No prognostic imbalances or differences in clinicopathological features were observed between the ITT and BM populations (Supplementary Figure 1 and Supplementary Table 1). Of the 1539 BM samples, nucleic acid was successfully extracted from 1181 tissue samples and subsequently profiled on the custom designed 800-gene expression panel. Eight hundred and sixty one samples had gene detection rate >75% (Supplementary Figure 2). Among 861 samples, 816 samples could be categorized into PAM50 subtypes with reasonable prediction confidence (prediction probability>0.4, Supplementary Figure 3). We identified 327 (40%) tumors as luminal A, 124 (15%) as luminal B, 69 (8%) as HER2-enrichded and 296 (36%) as basal-like. As expected, predicted genes and Ki67 were expressed in the appropriate subtypes (Supplementary Figures 4 and 5). For the basal and luminal subtypes, greater than 80% of the patients were classified as TNBC and HR^+^, respectively (Supplementary Figure 3). The most heterogeneous group was the HER2-enriched group in which only 41 out of the 69 classified as HER2-enriched were HER2^+^ (59.4%).

**Figure 1:**
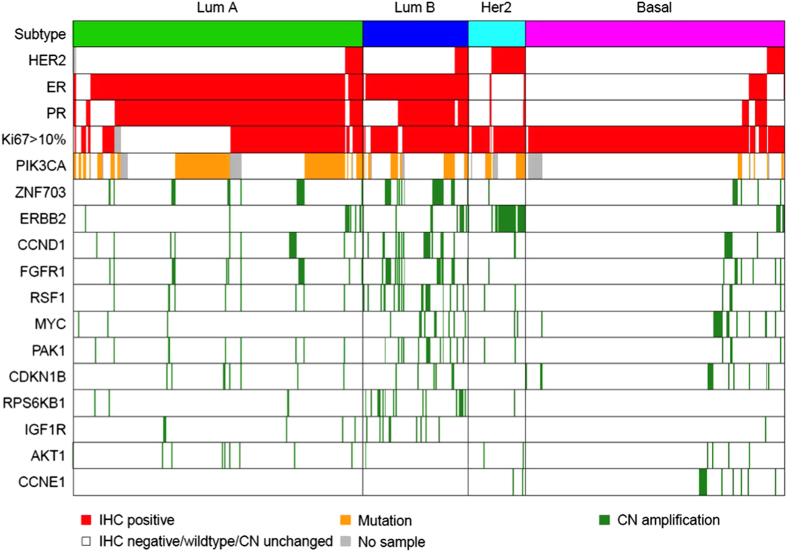
Molecular landscape of high-risk early breast cancer. Tumor samples are grouped according to intrinsic subtype; luminal A (*n*=285), luminal B (*n*=104), HER2-enriched (*n*=56), and basal-like (*n*=255). Red denotes IHC-positive cases, orange denotes *PIK3CA* mutation positive cases, green denotes copy-number amplified cases, white denotes wild-type or IHC-negative cases, and gray denotes no result available. IHC, immunohistochemical.

Of the 816 patients with the intrinsic subtype data, a subset of 700 tumor samples were also successfully run on the CNA panel (Figure 1). Luminal B tumors had higher expression of cell cycle and receptor tyrosine-kinase-related genes (e.g., *CCND1*, *FGFR1*, *IGF1R*, *MYC*) compared with luminal A tumors (Figure 1 and Supplementary Table 2). Cyclin E1 (*CCNE1*) was amplified in both the HER2-enriched and basal-like subgroups compared with the luminal subtypes (4% and 5%, respectively).

### Prognostic effects of the PAM50 intrinsic subtypes

**Figure 2:**
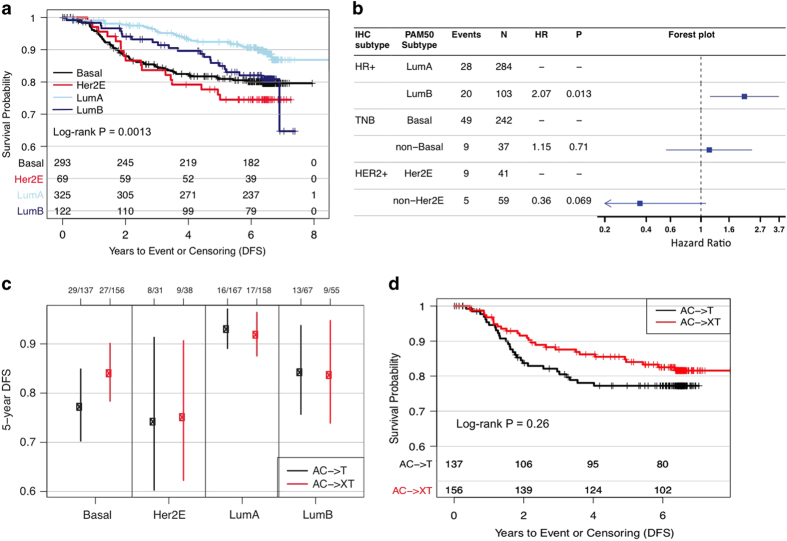
Effect of intrinsic subtypes on outcome. (**a**) Pooled arm analysis showing Kaplan–Meier curves of PAM50 status and DFS. (**b**) Forest plot depicting the effect of PAM50 status within IHC-defined subtypes. Her2E denotes HER2-enriched. In each subtype, the reported hazard ratio (HR) is calculated for DFS by comparing the two groups with the reference group being the top one. (**c**) Plot showing the effect of the addition of capecitabine to adjuvant chemotherapy with PAM50-defined subtypes. (**d**) Kaplan–Meier graph demonstrating the effect of capecitabine in the basal-like subgroup. A denotes doxorubicin, C denotes cyclophosphamide, T denotes docetaxel and X denotes capecitabine. DFS, disease-free survival; IHC, immunohistochemical.

As previously reported, the addition of capecitabine to adjuvant chemotherapy did not demonstrate a statistically significant effect on DFS after a 5-year follow-up.^20^ Given the low number of events overall (13%), we pooled the control and experimental arms to test the relationship of PAM50 intrinsic subtypes with prognosis for patients receiving adjuvant chemotherapy, and observed results consistent with those previously reported (Figure 2a). Of note, only ~30% of the HER2^+^ patients received adjuvant trastuzumab following chemotherapy when adjuvant trastuzumab became available, which likely explains the poor prognosis of the HER2-enriched subtype.

We found that 92.4% HR^+^ BC were determined to be either luminal A or B, 86.8% TNBC as basal-like and 40.6% HER2^+^ BC as HER2-like (Supplementary Figure 3), therefore we assessed the prognostic significance of the intrinsic subtype differences within IHC subtypes (Figure 2b). Within the HR^+^ IHC group, the luminal B subtype had a significantly worse DFS compared to the luminal A subtype (HR, 2.07; *P*=0.01). Within the TNBC IHC subgroup, no significant differences were observed between PAM50-predicted basal versus non-basal subtypes (HR, 1.15; *P*=0.71). Interestingly, within the HER2^+^ IHC subtype, the non-HER2-enriched subtype showed a marginally favorable prognosis compared to the HER2-enriched subtype (HR, 0.36; *P*=0.07). The non-HER2-enriched subtype included 23 HR^−^ and 36 HR^+^ tumors with no apparent differences in DFS observed (HR: 0.38 and HR: 0.35, respectively).

### Predictive effect for the addition of capecitabine to adjuvant therapy within the PAM50 intrinsic subtypes

As the addition of capecitabine to adjuvant therapy showed no statistical benefit across IHC subtypes,^20^ we evaluated whether there was any differential benefit from capecitabine within the PAM50 intrinsic subtypes. As shown in Figure 2c, no differences were seen within the HER2-enriched, luminal A or luminal B subtypes with the addition of capecitabine versus not. However, a non-significant increase in DFS was observed in the basal-like subtype with the addition of capecitabine to standard adjuvant chemotherapy (HR, 0.75; *P*=0.26, Figure 2d), which is similar to that observed in the TNBC population from the primary report (HR, 0.62; *n*=780).^20^

### Prevalence and prognostic effects of *PIK3CA* mutations

**Figure 3:**
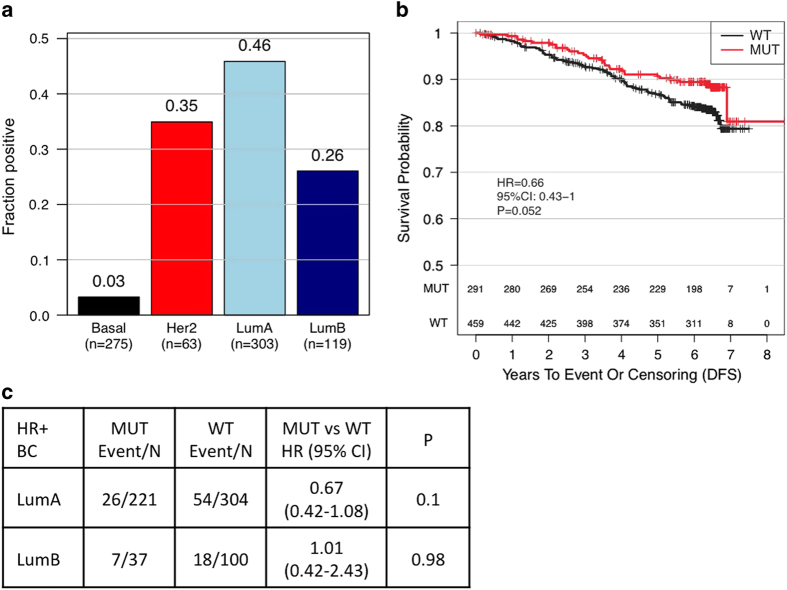
Prevalence and prognostic role of *PIK3CA* mutations. (**a**) Bar graph showing the prevalence of *PIK3CA* mutations within intrinsic subtypes. (**b**) Kaplan–Meier graph showing the prognostic role of *PIK3CA* mutations in HR^+^ breast cancer patients. (**c**) Univariate CoxPH model assessing the prognostic role of *PIK3CA* mutations within luminal A and B cancers. HR^+^, hormone receptor positive.

*PIK3CA* mutations were seen at a prevalence of 46% in the luminal A subtype, 35% in the HER2-enriched subtype, 26% in the luminal B subtype and 3% in the basal subtype (Figure 3a; Supplementary Figure 6a). Mutations in *PIK3CA* were found to be prognostic within the HR^+^ subset of patients (HR, 0.66; *P*=0.052; Figure 3b), with little outcome difference observed between exon 9 and exon 20 mutations (Supplementary Figure 6b). This effect appeared to be more driven by the Luminal A subset, although it did not reach statistical significance (Figure 3C, HR 0.67; *P*=0.1).

### Heterogeneity of TNBC tumors and response to treatment

**Figure 4:**
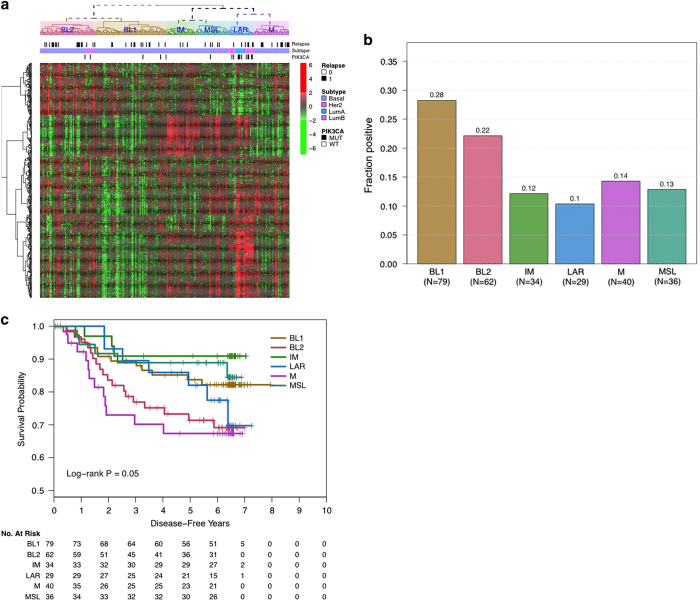
Prevalence and prognostic implications of molecular subtypes within TNBC. Heatmap (**a**), prevalence (**b**) and prognostic role (**c**) of the Lehmann *et al.*^15^ molecular subtypes within triple-negative breast cancer patients. PAM50-defined subtype and *PIK3CA* mutation status are indicated in (**a**). BL1, basal-like 1; BL2, basal-like 2; IM, immunomodulatory; LAR, luminal androgen receptor; M, mesenchymal; MSL, mesenchymal stem-like; TNBCs, triple-negative breast cancers.

TNBC is a heterogeneous subtype that can be molecularly subtyped using gene expression profiling.^15^ As demonstrated in Figure 4a,b, RNA profiling of the 280 triple-negative patients, defined by IHC, clustered patients into distinct subgroups based on a subset of the Lehmann *et al.* gene list. The basal-like (BL) 1 and 2 subgroups clustered closely and accounted for 28% and 22%, respectively, the immunomodulatory (IM) subgroup accounted for 12%, the mesenchymal-like (M) accounted for 14% and mesenchymal stem-like (MSL) subgroup accounted for 13%. Last, the luminal androgen receptor (LAR) accounted for 10% of the population and was largely predicted to be either HER2-enriched (55%) or luminal A (38%) based on PAM50 subtyping and accounted for the majority of *PIK3CA* mutations (65%) observed within the TNBC population (Figure 4a). Notably, distinct outcomes were observed in these subtypes with the M subgroup having the worst prognosis and the IM and MSL subtypes displaying the most favorable prognosis (Figure 4c).

### Immune gene profiling in adjuvant breast cancers

**Figure 5:**
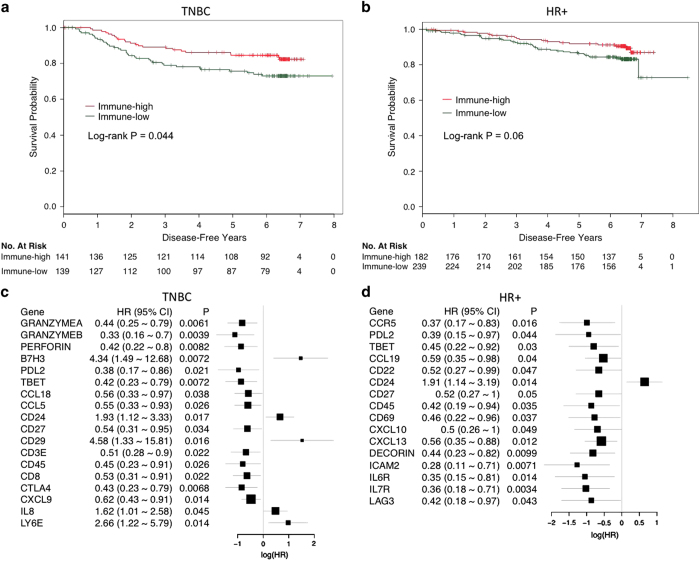
Role of immunologic and immune-related genes in TNBC and HR^+^ breast cancers. (**a**, **b**) Kaplan–Meier curves demonstrating the prognostic effect of the immune-high population (red) compared with the immune-low population (green) in TNBC (**a**) and HR^+^ breast cancer (**b**). (**c**, **d**) Forest plots showing the linear analysis of the significantly associated genes with clinical outcome, adjusted by treatment in both TNBC (**c**) and HR^+^ breast cancer (**d**). HR^+^, hormone receptor positive; TNBCs, triple-negative breast cancers.

Tumor lymphocyte infiltration has been demonstrated to correlate with pCR in the neoadjuvant setting.^16–19^ Supervised cluster analysis of 88 immunologic and immune-related genes found that immune-high and low populations could be identified in all subtypes of breast cancer (Supplementary Figure 7). Immune-high tumors correlated with improved DFS in the TNBC population (*P*=0.04, Figure 5a), showed a marginal improvement on DFS in the HR^+^ population and did not reach significance in the HER2^+^ population, likely due to limited sample size. Analysis of individual immune-related genes with outcome within the TNBC population revealed that genes involved in T-cell mediated cytotoxicity (e.g., *GZMA*, *PRF1*, *PDL2*, *CD8A*, and so on, Figure 5c) correlated with a better DFS outcome. Expression of five immune-related genes correlated with a poorer DFS outcome in TNBC patients (*B7H3*, *CD24*, *CD29*, *IL8*, and *LY6E*). Within HR^+^ breast cancer, higher expression of immunologic genes trended to have a more favorable DFS rate compared to tumors with low expression, although did not reach significance (*P*=0.06, Figure 5b). These genes were largely different to those identified in the TNBC tumors, in that genes associated with chemokine receptors (e.g., *CCR5*, *IL6R* and *IL7R*) and chemokine ligands (e.g., *CCL19*, *CXCL10* and *CXCL13*) correlated with a better clinical outcome (Figure 5d). Interestingly, we found that the genes *CD22*, *CD45*, and *CD27*, which are associated with B-cell activation and in mediating B-cell–B-cell interactions, also correlated with a better outcome in HR^+^ tumors. Similar to the situation in TNBC (HR 1.93, *P*=0.02), *CD24* expression correlated with a poorer outcome in HR^+^ cancers (HR 1.91, *P*=0.01).

### Identification of genes associated with risk of recurrence within 5 years in patients receiving adjuvant chemotherapy

**Figure 6:**
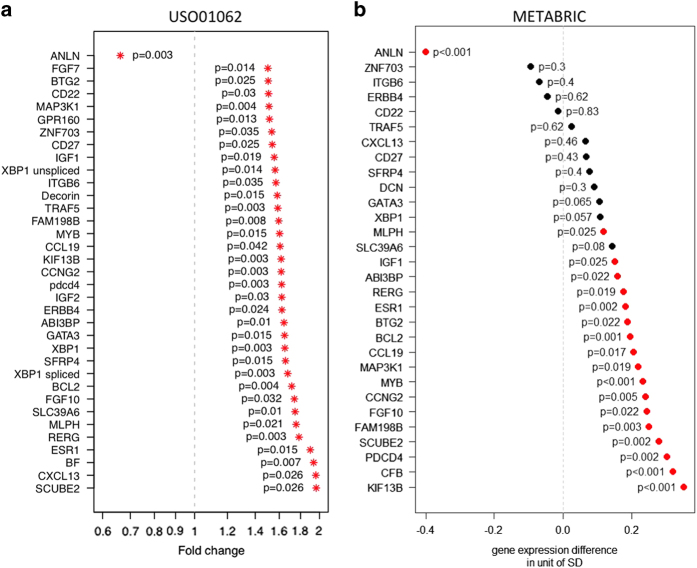
Genes associated with decreased 5-year DFS in HR^+^ patients. (**a**) Forest plot depicting the 35 genes associated with an increased risk of recurrence within 5 years in HR^+^ patients. (**b**) Validation of high-risk genes utilizing the METABRIC data set.^8^ Genes that were significantly associated with a 5-year disease-specific survival (DSS) are indicated in red. HR^+^, hormone receptor positive.

Identification of patients whose disease is most likely to recur within 5 years of adjuvant therapy represents a high unmet clinical need, since better understanding of this population could lead to more specific therapeutic interventions. We identified 35 genes whose expression was associated with a 5-year risk of recurrence in the HR^+^ population of greater than 1.5-fold difference between the high- and low-risk groups and a false discovery rate of 0.05 (Figure 6a). Of the 35 genes identified, low expression of only one gene (*ANLN*) was associated with a lower risk of recurrence, whereas higher expression of the remaining 34 genes correlated with a lower risk of recurrence. Not surprising, a subset of the genes identified (e.g. *ANLN*, *MLPH*, *BCL2*, *ESR1*, and *SCUBE2*) were associated with already known prognostic signatures, such as PAM50 and OncotypeDx.^6,21^ Next, to validate the prognostic role of the 35 genes, we utilized an independent data set from the publically available METABRIC project.^8^ Of the 35 genes identified in the training set, 18 were found to significantly associate with 5-year disease-specific survival (DSS) in the test set (Figure 6b). Similar to the training set, a proportion of the genes were associated with known prognostic signatures, however, several genes that are not previously reported in known prognostic signatures, including as *RERG*, *PCDC4*, *MAP3K1*, *CCL19*, and *ABI3BP*, were validated in the independent data set. We next examined the association of the 18 prognostic genes with a panel of 17 proliferation genes previously described to drive prognosis within the IHC subtypes.^22^ We did not observe a strong correlation between the 18 identified genes, with the exception of *ANLN*, which was highly correlated with all 17 proliferation genes (Supplementary Figure 8C). In the TNBC population, low expression of 5 genes were found to correlate with a higher 5-year risk of recurrence in the training set, but did not reach significance at the 5-year DSS endpoint in the validation set (Supplementary Figure 8).

## Discussion

Breast cancer is a heterogeneous disease generally accepted to have at least five distinct subtypes that can be defined by gene expression alone, and up to 10 subtypes when analyzed using integrated CNA and gene expression profiles.^6,8^ We molecularly characterized 861 samples from patients enrolled onto a phase 3 clinical trial that assessed the efficacy of adding capecitabine to standard adjuvant chemotherapy in high-risk early breast cancer patients. A key question in this patient population is the molecular definition of high-risk characteristics in order to identify patients who may benefit from cytotoxic therapy, and to identify predictors of poor prognosis that can be targeted therapeutically. Previous analyses of this patient population showed that patients whose cancers had low proliferation rates based on central Ki67 assessment had low recurrence rates and were less likely to benefit from the addition of capecitabine.^20^ Here we sought to use molecular analyses to more precisely define the high- and low-risk patients within this study population.

Intrinsic subtyping with the PAM50 algorithm suggested that the basal-like and HER2-enriched subtypes had the shortest DFS, consistent with results in other cohorts and likely reflecting the fact that most patients did not receive HER2-targeted therapies. We found that ~84% of the TNBC were classified as basal-like by PAM50 analysis, and that these patients showed a non-significant improvement in DFS with the addition of capecitabine. These results are similar to those from the FinXX study trial that showed the addition of capecitabine to adjuvant therapy improved recurrence-free survival in the TNBC population,^23^ and also from the ABCSG-24 study that demonstrated the addition of neoadjuvant capecitabine increased pCR rate in TNBC patients.^24^

Mutations within the *PIK3CA* gene are the second most common mutation in breast cancer.^12^ *PIK3CA* mutations in the basal subtype were largely confined to the LAR subtype, which has been previously reported to harbor a high prevalence of *PIK3CA* mutations.^25^ Within the luminal subtype, *PIK3CA* is the most commonly mutated gene and has been demonstrated to be a good prognostic factor in early stage disease.^26^ However, in a recent large adjuvant study, *PIK3CA* mutations were not found to be an independent prognostic factor in a multivariate analysis.^27^ As *PIK3CA* mutations are more common in the luminal A subtype, it is plausible that classifying tumors by *PIK3CA* mutation status may artificially select for a more favorable prognostic group. Interestingly, when controlled for intrinsic subtype, we found that the favorable prognosis associated with a *PIK3CA* mutation was only driven within the luminal A, but not in the luminal B subtype, although the difference did not reach statistical significance.

In addition to being characterized as basal or non-basal, TNBC have recently been defined further using RNA profiling by Lehmann *et al*.^15^ Furthermore, Masuda *et al.*, demonstrated the prognostic significance of these subtypes, suggesting that the IM and LAR subtypes had an increased rate of distant metastasis-free survival, whereas the BL2 and M subtypes had the lowest rate.^28^ We assessed representative genes (*n*=438) from the Lehmann signature, and were able to categorize TNBC into the published molecular subtypes. Similarly, we found that the IM subgroup had the most favorable DFS, whereas the M subtype had the worst. However, in contrast to the Masuda *et al.* findings, in our study the LAR subtype had a worse prognosis, whereas the MSL had a better prognosis, perhaps because the MSL subtype, like the IM subtype, expresses high levels of immune-associated genes.

Recently, Denkert *et al*.^16^ profiled 12 immunologic genes including T- and B-cell markers, chemokines and immune checkpoint modulators and demonstrated a strong correlation with TIL infiltration that was significantly associated with achieving pCR following neoadjuvant therapy in TNBC and HER2^+^ patients. Loi *et al.* ^18^ and Perez *et al.* ^29^ have recently showed that an activated immune response is associated with benefit from trastuzumab-based therapies in the adjuvant setting, suggesting that the association of TIL infiltration and pCR seen in the neoadjuvant setting will likely translate into improved DFS in the adjuvant setting. In our study, we show that increased expression of the T-cell mediated cytotoxicity genes *GZMA*, *GZMB*, and *PRF1* correlated with improved DFS in TNBC patients. We also show that the checkpoint-related genes *PDL2* and *CTLA4* correlated with an improved DFS, suggesting that TNBC’s primed for an activated T-cell response may benefit differentially from adjuvant chemotherapy. Interestingly, we observed a different immune signature in the HR^+^ population, identifying genes involved in cytokine and chemokine signaling as well as genes involved in B-cell activation and interaction that correlated with improved DFS, observations that warrant future investigations in independent studies. We also found that high expression of the adhesion molecule *CD24* correlated with poorer DFS in both TNBC and HR^+^ breast cancers. Expression of CD24 in preclinical mouse models has been shown to promote breast cancer metastasis by increasing both proliferation and adhesion.^30^ These results are timely given the fast paced development of cancer immunotherapy agents, as they suggest a role for such agents in early stage HR^+^ cancers in addition to HER2^+^ and TNBC.

Last, utilizing a discovery approach, we identified 18 genes associated with recurrence risk within 5 years in the HR^+^ population. For example, the Ras-like, estrogen-regulated, growth-inhibitor (*RERG*) gene, which has been previously shown to correlate with favorable biology in ER+ disease.^31,32^ The precise functional role of *RERG* is unknown, but expression of the protein has been shown to result in reduced growth and tumor formation in mice. Secondly, the tumor suppressor, programmed cell death 4 (*PDCD4*), has been shown to inhibit the translation machinery by binding to the translational initiation factor eIF4A. Decreased expression of *PDCD4* has been linked with resistance to aromatase inhibitors in preclinical models, while high expression is associated with a good outcome in HR^+^ disease.^33,34^ Thirdly, *MAP3K1*, a serine–threonine kinase, has been shown to mediate apoptosis through activation of the JNK proapoptosis protein.^35^ Interestingly, somatic inactivating mutations in *MAP3K1* are present in 8% of breast cancers, with luminal A tumors having a higher mutation rate than luminal B tumors.^10–12^

In conclusion, we molecularly characterized breast cancers obtained from high-risk patients enrolled onto a phase 3 adjuvant chemotherapy study. We found that patients with basal-like breast cancer had a trend towards increased DFS with the addition of capecitabine to adjuvant chemotherapy. We also show that TNBCs that displayed an activated T-cell cytotoxicity signature had a superior outcome compared with TNBCs that did not display the activated T-cell signature. Finally, we demonstrate that increased expression of *CD24* correlated with a worse DFS in both TNBC and HR^+^ breast cancers. Although retrospective in nature and requiring prospective validation in future studies, our findings suggest that molecular subtyping can be used to further stratify patients defined as ‘high-risk’ by clinical factors into distinct prognostic subgroups. A number of these subgroups have clinically actionable targets that could be tested as novel therapeutic interventions in neoadjuvant or adjuvant studies. Early breast cancer studies are challenging and take many years to complete due to generally low rates of disease recurrence. Future studies in this setting may benefit from enrolling high-risk patients based on molecular classification in addition to clinicopathologic parameters.

## Materials and methods

### USO01062 study

Patients were enrolled onto the parent study USO01062, A Randomized, Open-Label, Multicenter, phase Ill Trial Comparing Regimens of doxorubicin plus cyclophosphamide Followed by Either dodetaxel or dosetaxel plus capecitabine as Adjuvant Therapy for Female Patients with High-Risk Breast Cancer (clinicaltrials.gov/show/NCT00089479).^20^ Briefly, women aged ⩾18 and <70 years with high-risk (T1–3, N1–2, M0; or T>2 cm, N0, M0; or T>1 cm, N0, M0 and both ER- and PR-negative), operable, histologically confirmed adenocarcinoma of the breast were assigned to receive doxorubicin plus cyclophosphamide followed by docetaxel or doxorubicin plus cyclophosphamide followed by docetaxel plus capecitabine. Patients with HR^+^ disease received tamoxifen, an aromatase inhibitor, or both, sequentially, for 5 years. After 2005, patients with HER2- positive disease were offered 1 year of concurrent or post-study trastuzumab.

### Tissue collection

Formalin-fixed paraffin-embedded (FFPE) tumor samples were obtained from (*n*=1,539) breast cancer patients as part of the parent study. Tissue samples were collected and analyzed following approval by the US Oncology. Institutional Review Board and appropriate confirmation of written informed consent. ER, PR, and HER2 status was determined by local testing (Supplementary Appendix 1).

### Gene expression

Hematoxylin and eosin sections were prepared for all samples and were reviewed by a pathologist to confirm diagnosis and assess tumor content. RNA extraction and gene expression analysis was performed as previously described.^36^ Briefly, FFPE sections were macrodissected to enrich for neoplastic tissue followed by RNA extraction using the High Pure FFPE RNA Micro kit (Roche Applied Sciences, Penzberg, Germany). Gene expression was subsequently determined using the NanoString nCounter Analysis System (NanoString, Seattle, WA, USA) on a custom designed 800-gene panel tailored for breast cancer. Briefly, a total of 200 ng of RNA was hybridized to the codeset overnight at 65 °C according to the NanoString protocol. Samples were loaded onto the NanoString nCounter Prep Station and read using the NanoString nCounter Digital Analyzer. Raw expression data was then log2-transformed and normalized against included housekeeping genes (Supplementary Appendix 2).

Of the 1,539 BM samples originally used to measure Ki67 protein expression by IHC in the primary manuscript,^20^ DNA and RNA was extracted from the remaining tissues. One thousand, one hundred and eighty-one samples had sufficient RNA quantity and concentration (200 ng at 50 ng/μl) to profile using the custom designed 800-gene expression panel. Eight hundred and sixty one samples passed the QC metric of ⩾75% gene detection rate (Supplementary Figure 2), which was defined as the percentage of genes whose expression counts are larger than the 99.5% confidence interval of expression counts of 8 negative controls for a given sample. Of the 861 samples, 817 samples were further categorized into PAM50 subtypes with prediction confidence >0.4 (Supplementary Figure 3). RNA concentration of QC-failed samples was significantly lower than QC-passed samples (*P*<2.2e^−16^ (27 vs 49 ng/μl), Wilcoxon Rank Test). Comparing 260/280 ratio of 2 or greater, more samples with lower 260/280 ratio resided in the QC-failed group (odds ratio=6.38, *P*=4.5e^−11^, Fisher’s Exact Test). A marginal association between QC failure and tumor content between failed and passed samples (average tumor content: 29.7% vs 32.9% respectively), and also age of block (average age: 8.2 vs 7.9 years, respectively) was observed.

### *PIK3CA* mutation analysis

Five-micron sections were cut from each FFPE tumor block and DNA extracted using the QIAamp FFPE kit (Qiagen, Hilden, Germany) after deparaffinization with Envirene, as described previously.^36–38^ DNA (160 ng) from each sample was molecularly profiled using an internally developed multiplex PCR somatic hotspot mutation assay (MUT-MAP) that detects hotspot mutations in *PIK3CA* gene, as described previously.^39,40^ A list of detected mutations are listed in Supplementary Appendix 3.

### Copy-number alteration

Genomic FFPE DNA (200 ng) was subjected to 17 cycles of preamplification using pooled gene specific primers at 50 nmol/l each and Taqman Preamplification Master Mix (Life Technologies, Carlsbad, CA, USA) according to the manufacture’s protocol. The preamplified samples were diluted fivefold and qPCR was performed using Fluidigm (South San Francisco, CA, USA) 96.96 Dynamic Arrays on the BioMark (Fluidigm) system according to the manufacturer’s instructions. In brief, sample mix contains DNA, Taqman Gene Expression Master Mix (Life Technologies), DNA-binding sample loading reagent (Fluidigm) and EvaGreen dye (Biotium, Fremont, CA, USA). Assay mix contains gene specific primer pairs and sample loading reagent (Fluidigm). The Ct determination and melt curve analyses were carried out using the Fluidigm gene analysis software. Relative gene copy numbers were calculated using a two-way ANOVAR^41^ script to normalize sample inputs and assay variations, assuming the median copy number of all genes in all samples was 2. Assays were designed and tested with standard curve of human-blood-derived genomic DNA to have efficiency between 80 to 120% and similar Cts. The majority of genes on the panel had three assays per genes and final gene copy-number estimates are the average of all of the assays for each gene (Supplementary Appendix 4).

One thousand two hundred and forty eight samples were ran on the CNA assay that had 200ng DNA available and QC was performed, per manufacturers’ instructions. CNA data from 700 samples that had matched RNA expression data with prediction confidence >0.4 PAM50 subtype calls were included in the analysis.

### Molecular classification

PAM50 subtype prediction was carried out using a random-forest-based classifier that was derived from an independent training set of 157 breast cancer samples (data not shown) and 50 genes from the public PAM50 signature.^6^ We assigned PAM50 subtypes for the training samples based on consensus calls from both public nearest-centroid based PAM50 classification and hierarchical clustering approaches to reduce platform and population biases. Among them, 112 samples had consistent calls from both approaches and formed the final training set. A random-forest based classifier was then developed with an estimated out-of-bag error rate of 7.1% and applied to predict new samples.

TNBC subtypes were examined on 280 HER2-/ER-/PR- samples by hierarchical clustering with Pearson’s correlation distance and Ward’s linkage method. Four hundred thirty eight genes that are previously reported as differentially expressed in TNBC subtypes^15^ and present in the custom 800-gene panel were used for clustering. Six major sub-clusters were identified. To assign individual clusters to previously defined subtypes, we examined associations of mean expression profile of individual clusters with reported subtype-specific genesets^15^ by single-sample GSEA.^42^ Individual clusters were then assigned to the subtype with highest enrichment score.

### Analysis of immune-related genes with clinical outcome

HR^+^ or TNBC samples were selected based on HER2, PR and ER IHC status. Hierarchical clustering with 88 precompiled immunologic and immune-related genes was performed using Manhattan distance and Ward’s linkage method for HR^+^ or TNBC samples respectively. Association of immune-high and immune-low clusters with DFS was analyzed using Cox proportional hazards regression models and presented as Kaplan–Meier estimates with HR and 95% confidence interval. Statistical significance was assessed by a two-sided log-rank test and further adjusted for multiple testing by the Benjamini–Hochberg method in individual gene analysis. All analyses were carried out in R 3.2.

### Identification of genes associated with a high-risk population

We compared expressions of individual genes from the custom 800-gene expression panel between patients with DFS ⩽5 years and patients with DFS>5 years, within the subgroup of HR^+^/HER2- patients (*n*=49 and *n*=342 for DFS⩽5 years and DFS>5 years, respectively), and within the subgroup of triple-negative patients (*n*=54 and *n*=209 for DFS⩽5 years and DFS>5 years, respectively). A two-sample *t*-test is used to compare the log-transformed expression level of each gene in each subgroup. Regression diagnostics were performed to investigate normality and homoscedasticity, and normal distribution and similar variances were observed between groups for most of the genes (data not shown). The Benjamini–Hochberg procedure^43^ was used to adjust for multiple comparisons by controlling the false discovery rate. Genes with a mean fold-change in expression>1.5 or<0.67 and a Benjamini–Hochberg adjusted *P *value (false discovery rate)<0.05 were considered significant.

To validate our findings, expression profiling of genes from the METABRIC study was used as an independent validation set.^8^ For genes that were found significantly differentiated between patients with DFS⩽5 years and DFS>5 years in our study, we examined the gene expression levels between patients with DSS⩽5 years and patients with DSS>5 years within the corresponding patient subgroup (HR^+^ or TNBC) in the METABRIC data set. Similarly, two-sample *t*-test and Benjamini–Hochberg adjustment were used and genes with an adjusted *P *value<0.05 were considered significant.

### Code availability

All analyses were carried out in R 3.2 using custom scripts (available upon request).
