# Supplementary material for: The molecular landscape of high-risk early breast cancer: comprehensive biomarker analysis of a phase III adjuvant population
Source: NPJ Breast Cancer. 2016 Jul 13;2:16022–. doi: 10.1038/npjbcancer.2016.22 (PMC5515335; doi:10.1038/npjbcancer.2016.22)
Supplement: Supplemental Figures [file npjbcancer201622-s1.pdf]

Supplemental Figure 1

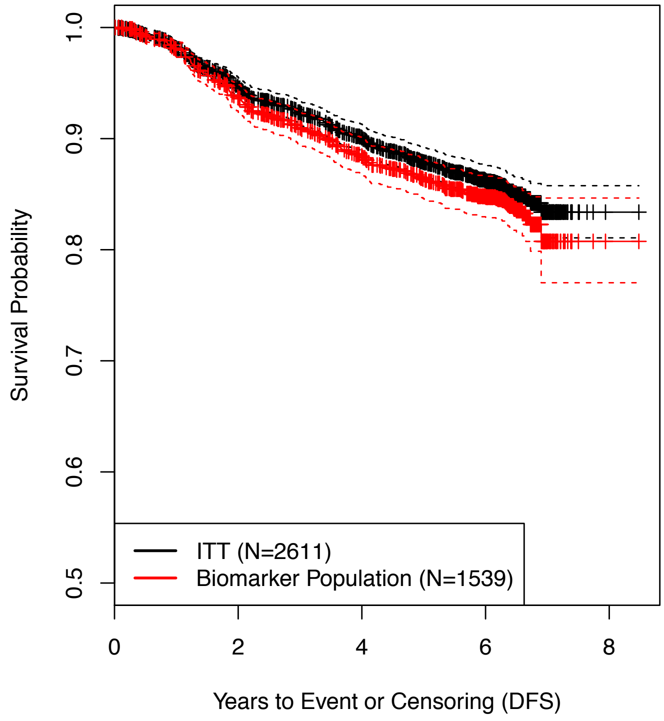

Supplemental Figure 1. Kaplan-Meier curves showing the intent to treat population (ITT) and the biomarker evaluable population.

Supplemental Figure 2

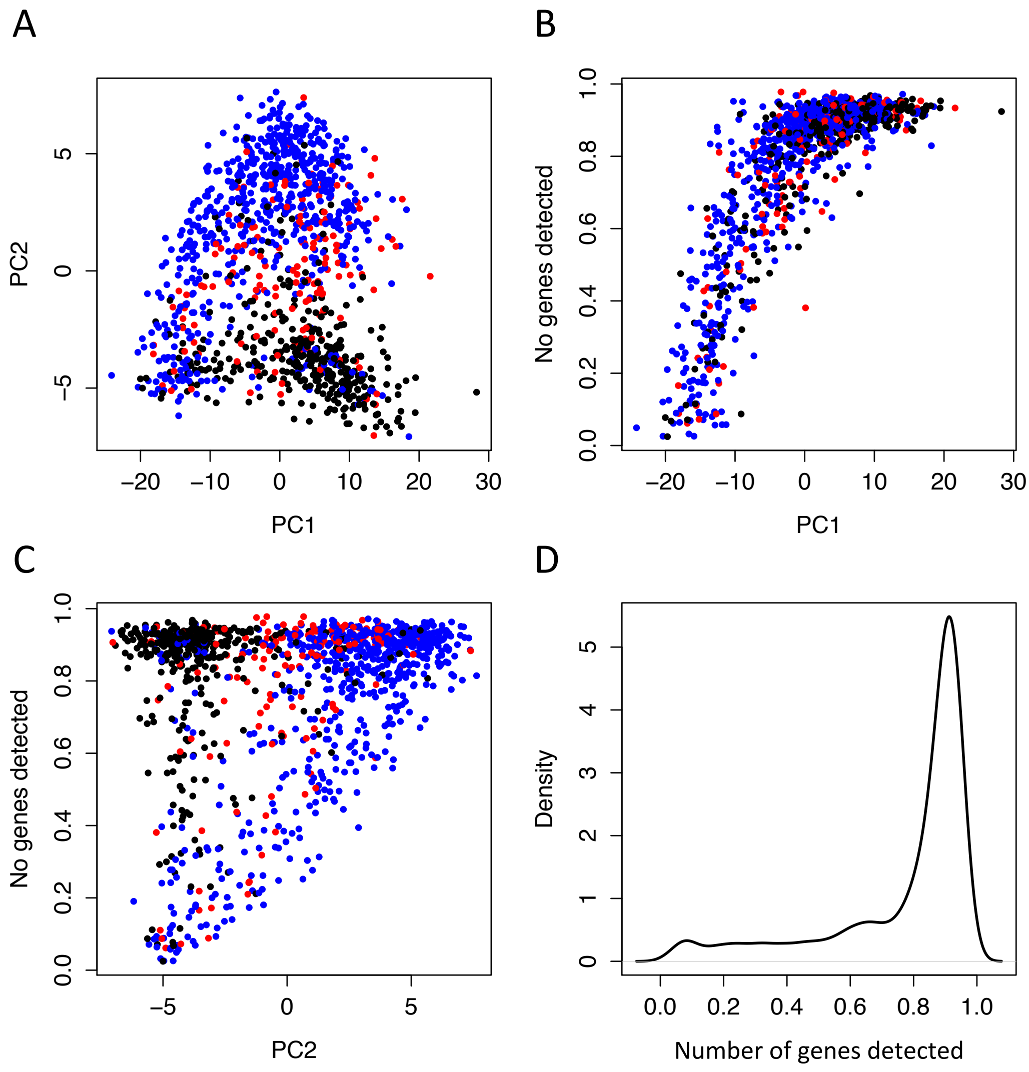

Supplemental Figure 2. Principal component analysis (PCA) of the 1181 samples run on the custom 800-gene expression panel. IHC-based subtypes are represented in different colors (red: HR+, black: TNBC, blue: HER2+). Distribution of gene detection rate across samples was shown in panel D. A gene is defined as 'detected' in a sample when its count is larger than the 99.5% confidence interval of the 8 negative controls for that sample. 861 samples passed the QC metric of greater than or equal to 0.75 gene detection rate.

Supplemental Figure 3

A

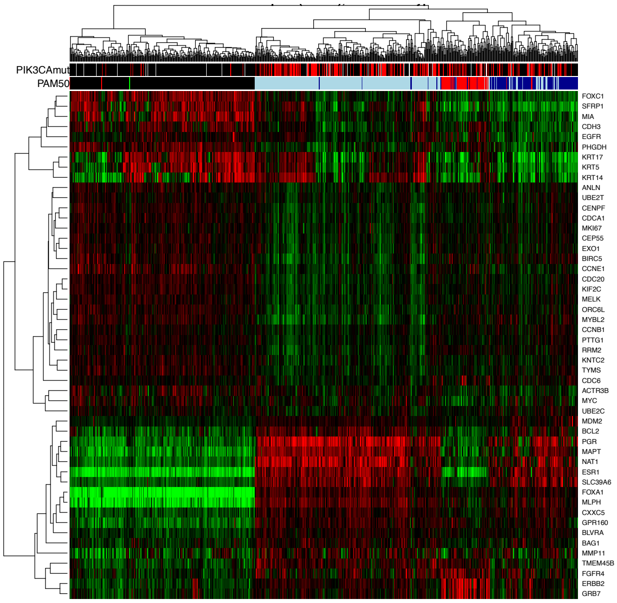

B

subtype	n	%
Basal-like	296	36.2
HER2-enriched	69	8.4
Luminal A	327	40.0
Luminal B	124	15.2
Normal-like	1	0.1

C

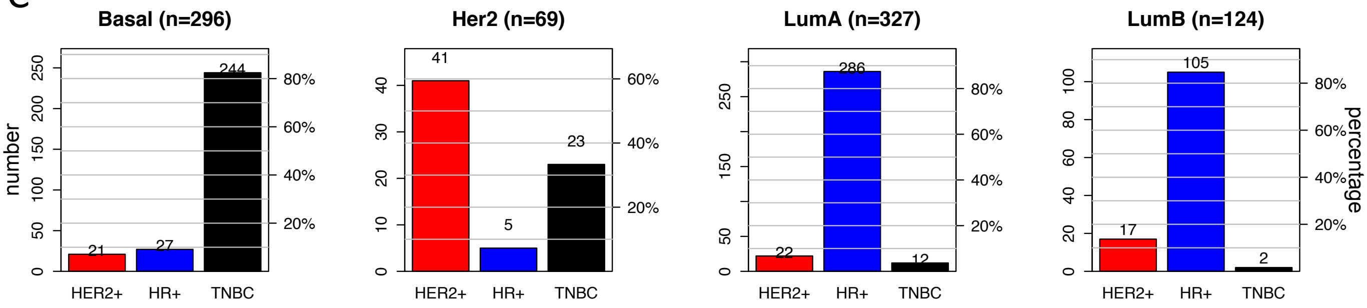

Supplemental Figure 3. A. Heatmap depicting the 817 QC passed samples using the PAM50 genes for intrinsic molecular classification. B. Table showing the number of samples in each PAM50 classification. C. Bar graphs indicating the IHC subtype within each PAM50 defined intrinsic subtype.

Supplemental Figure 4

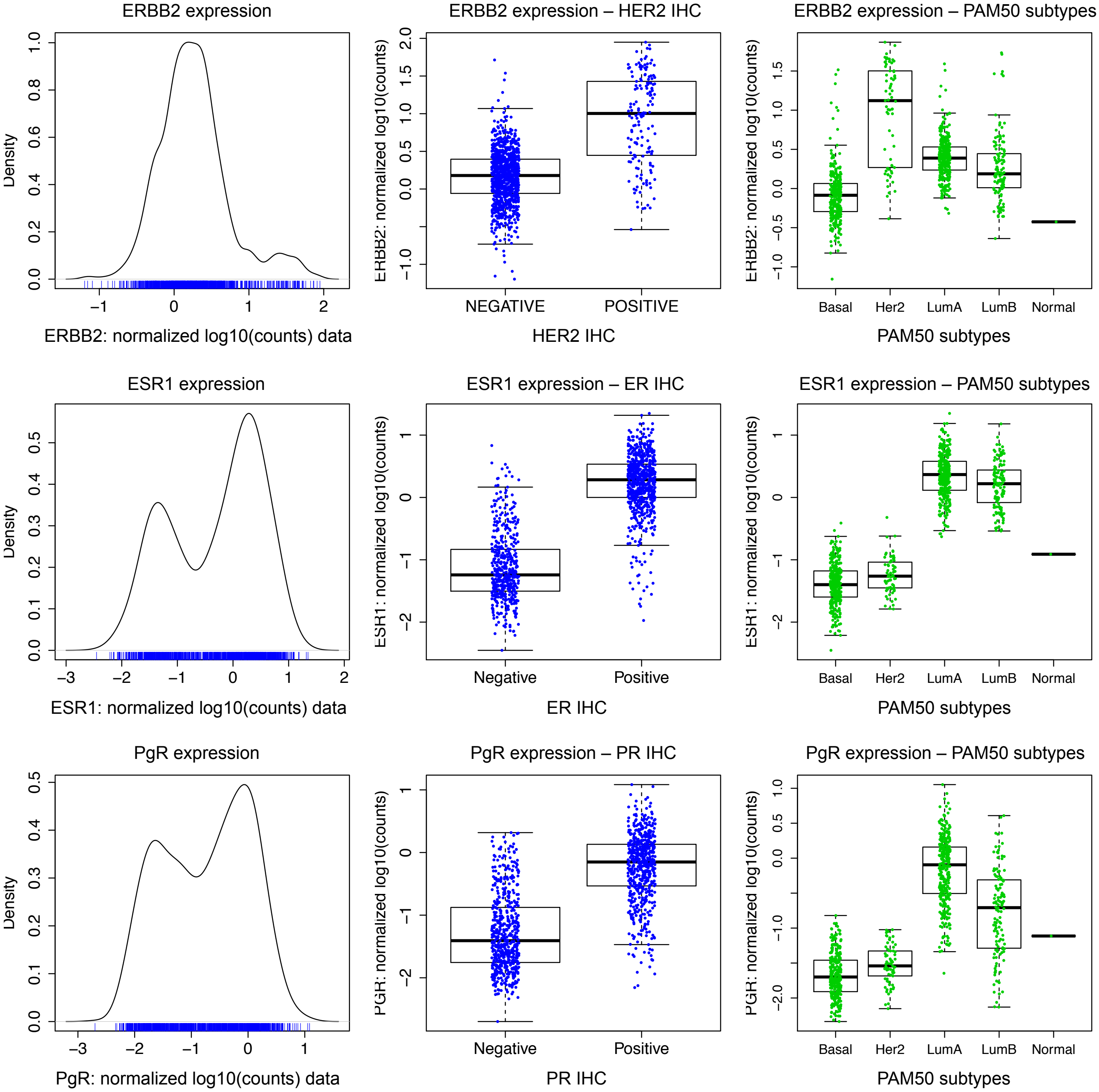

Supplemental Figure 4. Target gene expression within both IHC and PAM50-defined subtypes.

Supplemental Figure 5

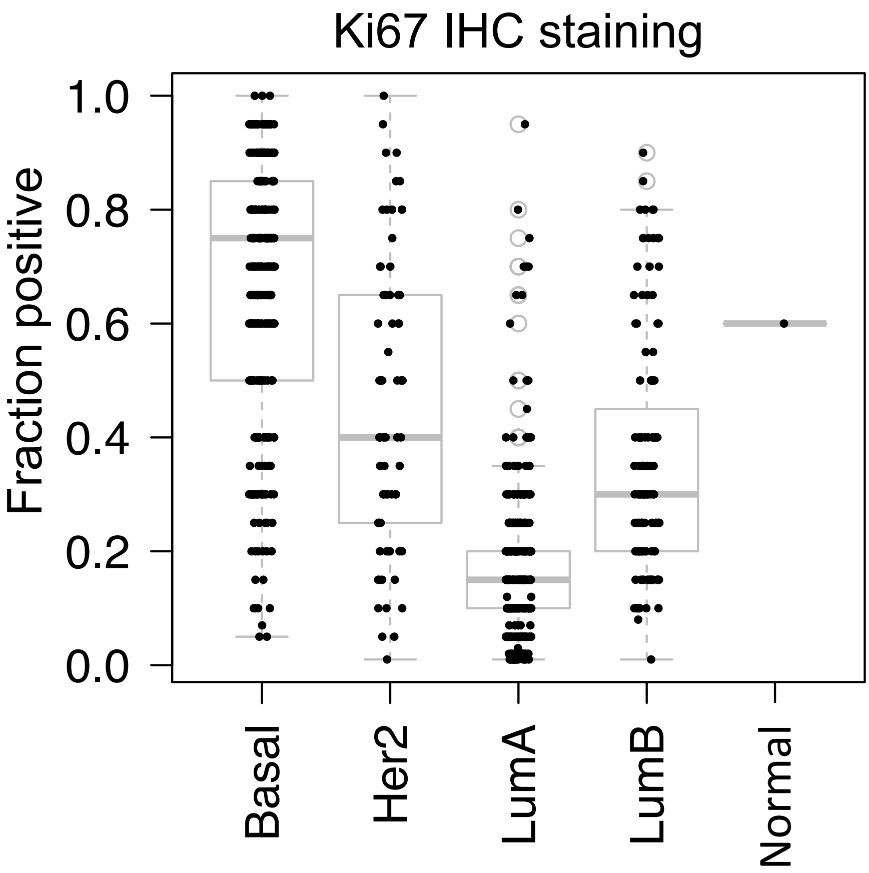

Supplemental Figure 5. Ki67 IHC staining within PAM50-defined subtypes.

Supplemental Figure 6

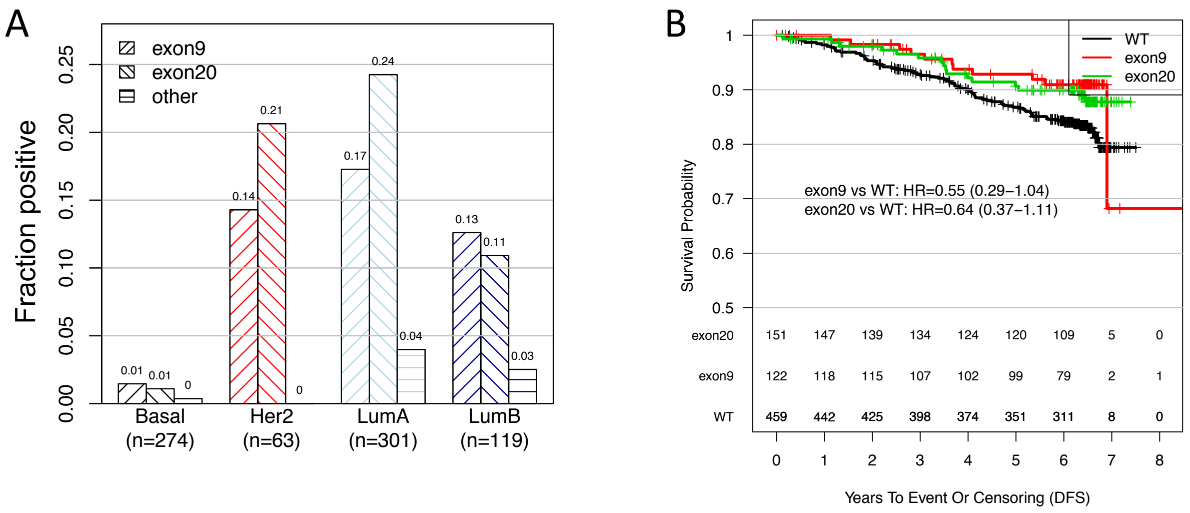

Supplemental Figure 6. A. Fraction of *PIK3CA* mutant tumors analyzed by exon in PAM50-defined subtypes. B. Kaplan-Meier showing the prognostic effect of exon 9 or exon 20 *PIK3CA* mutations in HR+ breast cancer patients.

Supplemental Figure 7

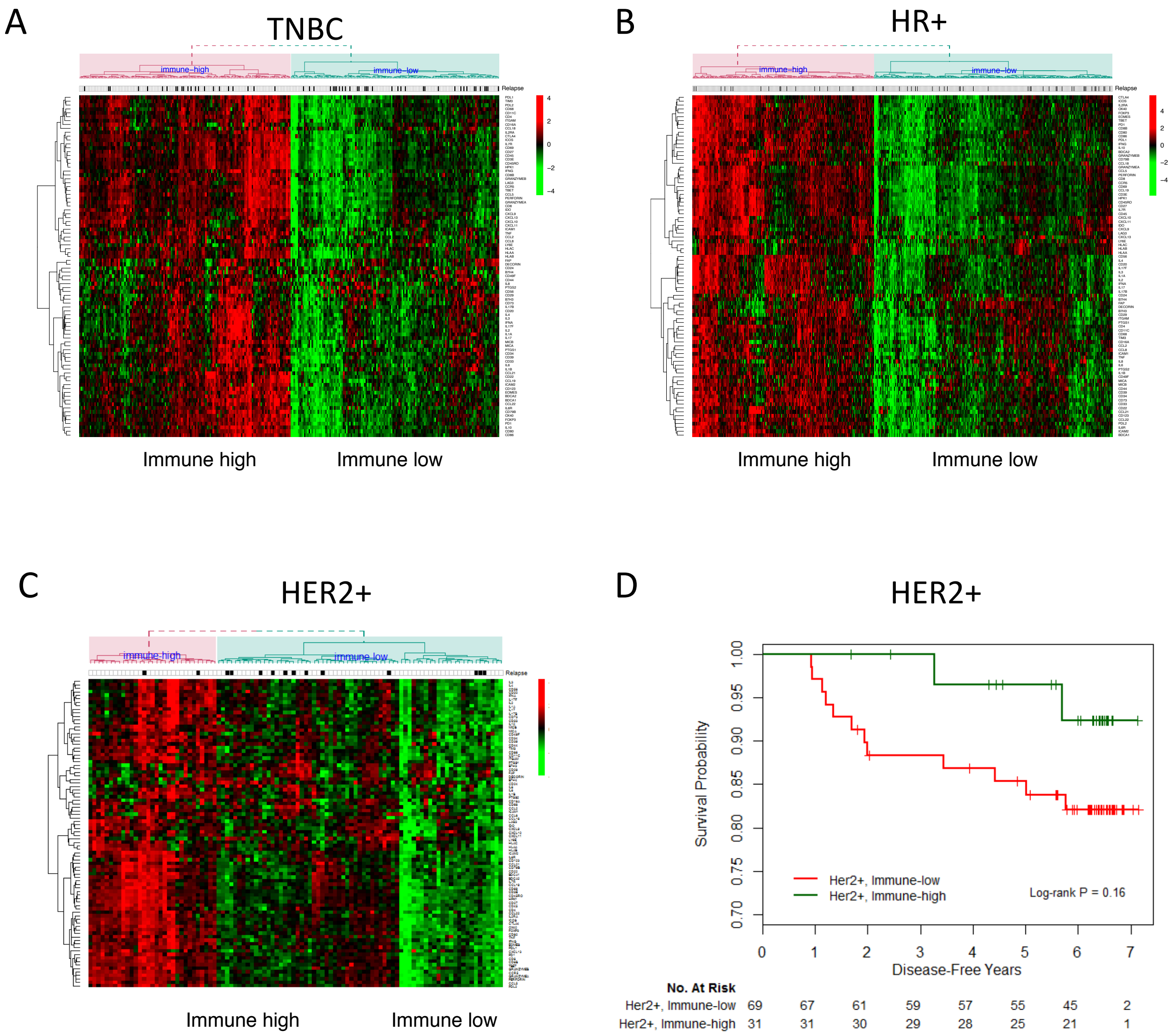

Supplemental Figure 7. Heatmap depicting the expression of immunologic and immune-related genes (n=88) within TNBC (A), HR+ (B) and HER2+ (C) breast cancer. D. Kaplan-Meier curves demonstrating the prognostic effect of the immune-high population (red) compared to the immune-low population (green) in HER2+ breast cancer

Supplemental Figure 8

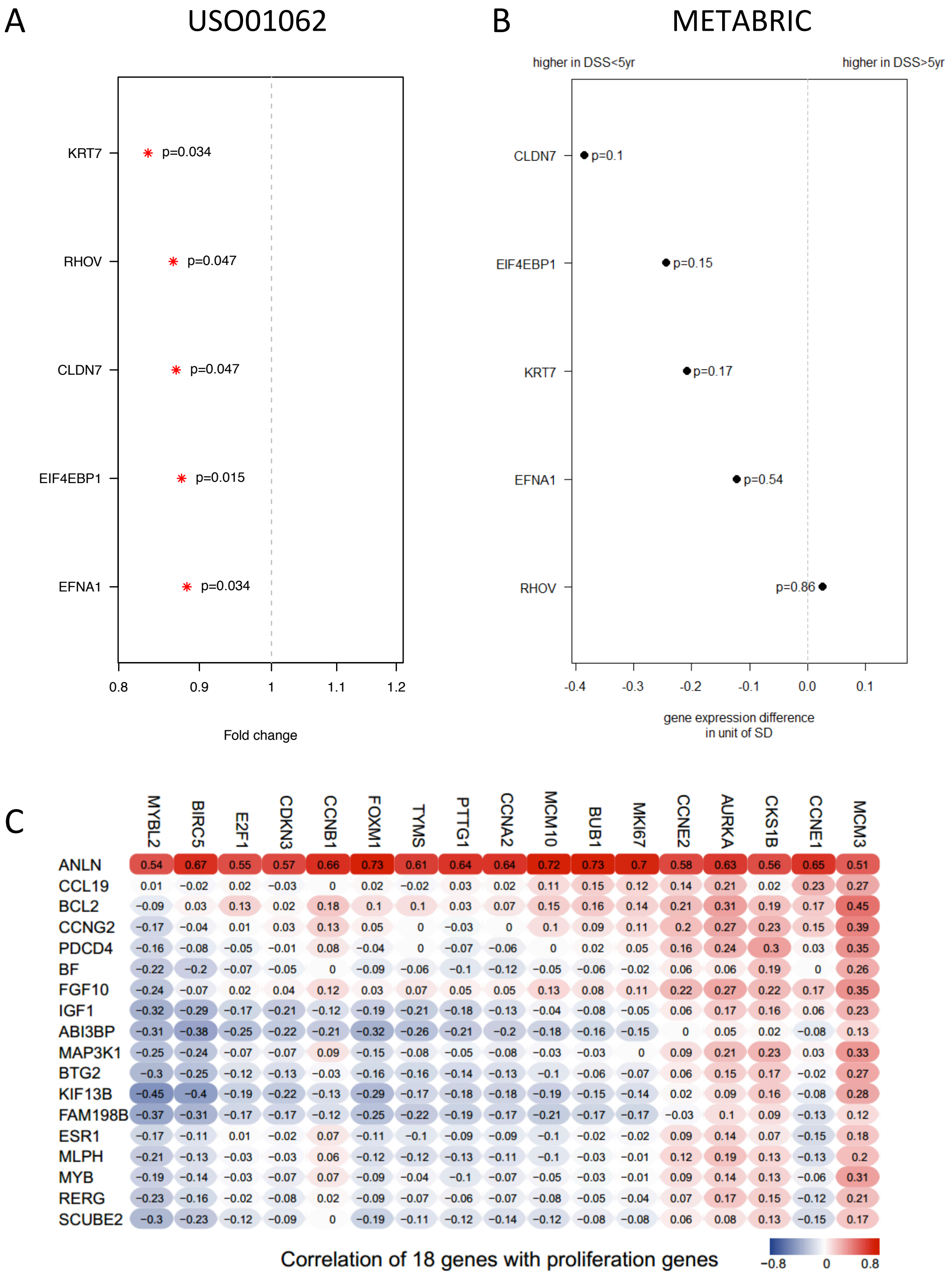

Supplemental Figure 8. A. Forest plot depicting the 5 genes associated with an increased risk of recurrence within 5 years in TNBC patients. B. Confirmation of high-risk genes with the METABRIC dataset. Genes that were significantly associated with 5-year disease specific survival are indicated in red. C. Correlation of the 18 identified HR+ high-risk genes with a panel of 17 proliferation genes (22).
