# Supplementary material for: The molecular landscape of high-risk early breast cancer: comprehensive biomarker analysis of a phase III adjuvant population
Source: NPJ Breast Cancer. 2016 Jul 13;2:16022–. doi: 10.1038/npjbcancer.2016.22 (PMC5515335; doi:10.1038/npjbcancer.2016.22)
Supplement: Supplementary Information [file npjbcancer201622-s2.doc]

|  | ITT | | | Biomarker Population | | |
| --- | --- | --- | --- | --- | --- | --- |
|  | Total | AC->XT | AC->T | Total | AC->XT | AC->T |
| N | 2611 | 1304 | 1307 | 1539 | 771 | 768 |
| Age |  |  |  |  |  |  |
| Median (Range) | 51 (26-72) | 50 (26-72) | 51 (26-70) | 51 (26-70) | 51 (26-70) | 51 (27-60) |
| Race |  |  |  |  |  |  |
| Caucasian | 2127 (81%) | 1052 (80%) | 1075 (82%) | 1259 (82%) | 616 (80%) | 643 (83%) |
| Lymph Node |  |  |  |  |  |  |
| Positive | 1819 (70%) | 915 (70%) | 904 (69%) | 1104 (72%) | 549 (71%) | 555 (72%) |
|  |  |  |  |  |  |  |
| Postmenopausal | 1454 (56%) | 721 (55%) | 733 (56%) | 871 (57%) | 435 (57%) | 436 (57%) |
| Stage |  |  |  |  |  |  |
| T1 | 976 (37%) | 492 (38%) | 484 (37%) | 579 (38%) | 297 (39%) | 282 (37%) |
| T2 | 1445 (55%) | 732 (56%) | 713 (55%) | 850 (55%) | 429 (56%) | 421 (55%) |
| T3 | 185 (7%) | 82 (6%) | 103 (8%) | 108 (7%) | 42 (5%) | 66 (9%) |

**Supplemental Table 1.** Clinicopathological features of intent to treat (ITT) and biomarker populations.


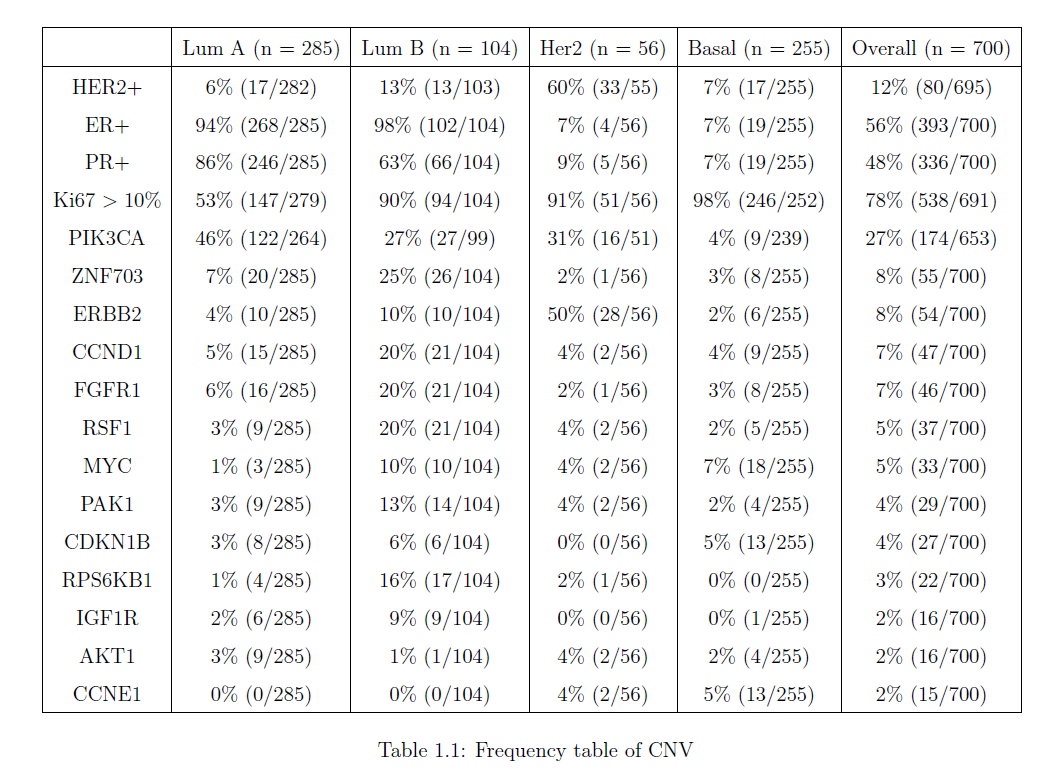


**Supplemental Table 2.** Biomarker prevalence within intrinsic subtypes.

**APPENDIX 3.** *PIK3CA* mutation coverage

| **Mutation** | **Exon** |
| --- | --- |
| R88Q | Exon 1 |
| N345K | Exon 4 |
| C420R | Exon 7 |
| E542K, E545A/D/G/K, Q546K/R/E/L | Exon 9 |
| M1043I/L, H1047L/R/Y, G1049R | Exon 20 |
